# Lime Treatment of Coal Bottom Ash for Use in Road Pavements: Application to El Jadida Zone in Morocco

**DOI:** 10.3390/ma12172674

**Published:** 2019-08-22

**Authors:** Souad El Moudni El Alami, Raja Moussaoui, Mohamed Monkade, Khaled Lahlou, Navid Hasheminejad, Alexandros Margaritis, Wim Van den bergh, Cedric Vuye

**Affiliations:** 1 Applied Geoscience Laboratory (LGA), Department of Geology, Mohamed First University, 60000 Oujda, Morocco; 2 Department of Physics, Chouaib Doukkali University, 24000 El Jadida, Morocco; 3 Department of Roads and Bridges, Hassania School of Public Works, 20000 Casablanca, Morocco; 4 Energy and Materials in Infrastructure and Buildings (EMIB) Research Group, Faculty of Applied Engineering, University of Antwerp, 2020 Antwerp, Belgium

**Keywords:** coal bottom ash, lime treatment, modified Proctor, CBR, subbase, road construction

## Abstract

**Featured Application:**

**In this paper waste bottom ash from a thermal power station was treated with lime and sand. This improves certain material characteristics leading to a potential use in a subbase in road construction.**

**Abstract:**

Industrial waste causes environmental, economic, and social problems. In Morocco, the Jorf Lasfar Thermal Power Station produces two types of coal ash with enormous quantities: fly ash (FA) and Bottom ash (BA). FA is recovered in cement while BA is stored in landfills. To reduce the effects of BA disposal in landfills, several experimental studies have tested the possibility of their recovery in the road construction, especially as a subbase. In the first phase of this study, the BA underwent a physicochemical and geotechnical characterization. The results obtained show that the BA should be treated to improve its mechanical properties. The most commonly used materials are lime and cement. In the selected low-cost treatment, which is the subject of the second phase of the study, lime is used to improve the low pozzolanicity of BA while calcarenite sand is used to increase the compactness. Several mixtures containing BA, lime, and calcarenite sand were prepared. Each of these mixtures was compacted in modified Proctor molds and then subjected to a series of tests to study the following characteristics: compressive strength, dry and wet California Bearing Ratio (CBR), dry density and swelling. The composition of each mixture was based on an experimental design approach. The results show that the values of the compressive strength, the dry density, and the CBR index have increased after treatment, potentially leading to a valorization of the treated BA for use in a subbase.

## 1. Introduction

The fast expansion of infrastructure in developing countries, such as Morocco, increases the need for raw materials. In addition, the continued production of waste, in particular solid waste, requires the implementation of a waste storage system. This storage causes several economic, urban, and environmental problems. Therefore, there have been multiple studies over the possibility of using these by-products in the field of construction, including road construction [1,2]. In past years, different types of waste such as coal ash, steel slag and municipal solid waste incinerator ash [1,3,4] and other more recent waste types such as silicon waste [5] have been investigated in order to test the possibility of their valorization in civil engineering applications. 

Coal ash, which is a residue of coal combustion in thermal power plants, has been the subject of several research projects, distinguishing between fly ash and bottom ash [1,2]. Fly Ash (FA) is a fine residue which is recovered at the level of the electrofilters and then sent to storage hoppers. They have been used for years as a substitute for cement in the field of construction [6]. On the other hand, the Bottom Ash (BA) has a sandy appearance [2]. It should be noted that the BA is recovered in a wet state (after cooling under water) at the bottom of the boiler.

The coal ash characterization work carried out by several researchers has shown the possibility of their use in the field of infrastructure and construction [1,2]. For instance, the BA has been used as a substitute for aggregates for the production of the bituminous mix [1]. However, the use of BA in asphalt mixtures decreases some mechanical properties such as tensile strength. To solve this issue, lime was used in the mixtures, which was able to significantly improve these mechanical properties [1]. BA has also been used in concrete as a partial substitute for sand [7]. Although, this causes a decrease in compressive strength. For example, for a concrete containing 10% BA as a sand substitute, the decrease in compressive strength exceeds 10% compared to conventional concrete. This decrease is attributable to the higher porosity of the BA, which causes a higher demand for water [2]. To solve this problem, a super plasticizer was added, which allows the reduction of the water demand and leading to an increase of the compressive strength [8]. BA was mixed with ordinary Portland cement and even rice husk ash in [9] to develop self-leveling hybrid mortars. In [10] a mixture of soil, BA, FA, crumb rubber, cement and water was investigated as flowable backfill material for an underground pipeline. Finally, geopolymers including BA have been investigated as well [11,12,13]. The actual performance of the geopolymer-based materials is strongly dependent on the specific local materials (mix of FA and BA), processing conditions and used blend.

Jorf Lasfar Energy Company (JLEC) is an energy company located in El Jadida, Morocco. JLEC consumes 5.4 million tons/year of coal to deliver more than 40% of Morocco’s electricity demands. This generates a large amount of ash (both FA and BA). FA accounts for 80% of the solid residues produced, an annual average exceeding 470,000 tons. As for the BA, they constitute 20% of the solid residues and represent more than 40,000 tons/year [6,14]. The majority of FA is used in Moroccan cement, while BA is still stored in landfills [6,14]. In addition to the economic burden, in terms of transport costs and storage of these residues, there is an environmental risk in terms of leaching of the elements, polluting the water underground. Therefore, an alternative solution to the landfill is investigated. As shown in [15,16,17] solidification/stabilization (S/S) can be considered as an effective action to prevent and minimize the release of contaminants into the environment.

This research focuses on an experimental study of JLEC Bottom Ash to test the possibility of their reutilization in the foundation layer or subbase of the road. First, the BA has undergone a complete characterization to determine the following properties: particle size, cleanliness, density, pozzolanic activity, and both chemical and mineralogical composition. Following the BA characterization results, it was suggested to treat BA with lime (*L*) and sand (*S*). The percentage of lime varies from 1 to 5% of the total weight of the mixture while that of sand is between 5 and 25%. The water (*W*) content of the mixture is between 21 and 22.4%. For each mixture, cylindrical specimens were compacted in a modified Proctor mold. In order to have reliable and statistically representative results, all the tests are performed with three repetitions, leading to a total number of test specimens of 192. The material properties (responses) that were tested are: compressive strength (*CS*), dry and wet California Bearing Ratio (*CBR_d_, CBR_w_*), dry density (*γ*), and swelling (*G*). For the design of the experiments the Design-Expert software was used. This makes it possible to define each characteristic output (response *Y_i_*) according to the influencing input factors (*X_i_*).

## 2. Materials and Methods

### 2.1. The Origin of the Materials

The BA is obtained from the JLEC plant EL Jadida, Morocco. They have been water-cooled and, therefore, they are recovered at the outlet of the boiler in a very humid (submerged) state, comparable to the moisture state of sea sand at the time of its extraction. Before being transported to the laboratory by truck (quantity necessary for all our tests), they were left outside the plant for three weeks in order to reduce their high water content sufficiently. Next, smaller quantities for each test batch were dried at the road geotechnical laboratory at the Hassania school of public works (Casablanca, Morocco).

To evaluate the pozzolanic reactivity of BA, mortar molds containing cement, sand, and water were prepared. Next, BA was used as a partial substitute for standardized sand. The compressive strength (CS) of the specimens containing the BA was compared with those of the conventional sand-based mortar. The cement used is the CPJ45 produced by the Holcim Lafarge Group in Morocco. Its chemical composition is given in Section 3.1.

As will be presented in the results, the BA has a porous structure, low compactness, and low pozzolanic reactivity. These weak characteristics do not allow for their use in a road foundation layer. To improve their performance, the BA was treated with lime and calcarenite sand. We note that the choice of these two treatment materials is made, taking into consideration the results of similar research [1]. They have proven the effectiveness of these two materials in improving the properties of BA. Lime is used to improve the pozzolanic activity of BA, while calcarenite sand increases its compactness. The lime used is produced at the Tetouan plant of the Lafarge group in Casablanca (Morocco). Its chemical composition is marked by a free lime (CaO) content greater than 80% whereas that of MgO does not exceed 8%. For the sand, we used a granular class 0/3.

### 2.2. Methodology

**Figure 1:**
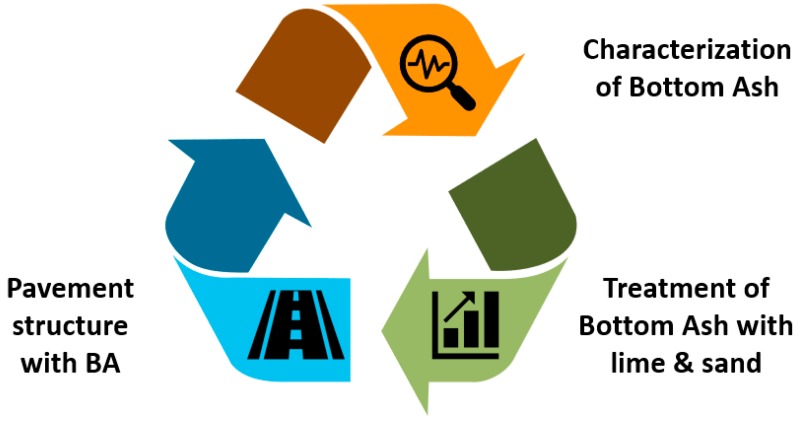
Methodology of this study comprising of three phases: characterization, treatment with lime and sand, and implementation in the theoretical design of a pavement structure.

This work was carried out in three phases, summarized as follows: the first phase of this study consists of a complete characterization of the BA. The characterization tests cover density, chemical and mineralogical composition, cleanliness, granulometric analysis, Proctor test, and wet and dry CBR indices. The second part describes the experimental program including results obtained for the treatment of the BA using lime and sand. In this part, we also explain the experimental design, obtained by the Design-Expert software [18]. Lastly, the third phase of this study consists of the theoretical design of a pavement structure, with and without BA treated with lime and sand. The design is done according to the classical method of the 1998 new pavement type structure catalogue [19]. This method takes into consideration: traffic, climate and geotechnical environment of the El Jadida area in Morocco, which represents the location envisaged for the future pavement. The used methodology is shown in Figure 1.

### 2.3. Characterization Methods

To test the possibility of using the BA in a foundation layer, several physicochemical and mechanical tests were carried out. The BA particles have a very porous shape. Their density is determined by the technique of the graduated cylinder [20], which consists in introducing a quantity of bottom ash in a known volume of water. The density is thus obtained by determining the volume occupied by this mass. The chemical composition of the BA Section 3.1 is obtained using X-ray fluorescence spectroscopy, while the mineralogical composition Section 3.1 is established using X-ray diffraction.

These two tests were carried out at the chemistry department at the Faculty of Sciences of the University of Hassan II in Casablanca, Morocco.

Next, the cleanliness of the BA is evaluated by two methods: sand equivalent (SE) method and methylene blue method according to the standards NF P18-598 [21] and NF P94-068 [22]. The first test gives us an idea of the rate of the clay part in our sample, the second determines the activity of this clay part and the degree of its sensitivity to water [23]. For the particle size analysis, the BA of class 0/20 mm was sieved according to the NF P18-560 standard [24], using 22 sieves from 0.04 up to 20 mm. The results obtained are compared to those specified by the ASTM standard [25] for BA used as a sand substitute [23]. In order to investigate the possibility of using BA in a road structure, the optimal compaction conditions, puncture resistance and swelling have been evaluated. In order to determine the optimal compaction conditions, the BA is sieved with a sieve of 20 mm and then subjected to a compaction test in a modified Proctor mold. The puncture resistance is measured twice: once compacted at natural water content as the dry CBR test (*CBR_d_*) and then after immersion for four days, called the wet CBR or CBR after immersion (*CBR_w_*). *CBR_d_* characterizes the ability of BA to withstand traffic flow. The swelling test is used to evaluate the behavior of BA under unfavorable humidity conditions. The swelling is measured during four days of immersion in order to test the volume stability of the BA [26]. The experimental protocols describing Proctor, CBR (dry and wet) and swelling test are detailed in the NF P94-078 standard [27].

**Figure 2:**
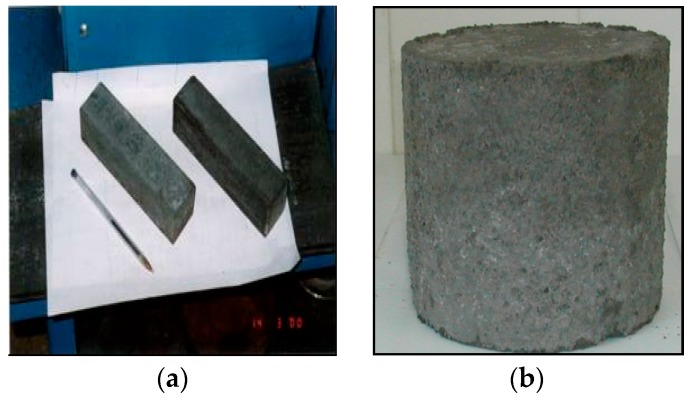
(**a**) Mortar prism (4 × 4 × 16 cm) (**b**) Compacted test tubes of bottom ash (*R* = 7 cm, *h* = 15 cm).

Finally, the pozzolanic activity was judged. A material is pozzolanic if it can, in the presence of moisture, chemically react with calcium hydroxide to form compounds with binding properties. To evaluate the pozzolanic activity of BA, three series of mortar prisms of dimension 4 × 4 × 16 mm were prepared in the building materials laboratory of the Hassania School of Public Works (Casablanca, Morocco), see Figure 2a. The production procedure for mortars is described in the Moroccan standard NM 10.1.004 [20] for hydraulic binders. The first series is the standard mortar, containing 450 g of cement, 1350 g of standardized sand and 225 g of water, which gives three mortar prisms. In the second and third series, part of the normalized sand mass is replaced by BA of size 0/2 mm with percentages of respectively 25% and 50%. The prepared mixtures are introduced into the mortar molds, which will be stored in a wet cupboard at a temperature of 20 °C. After 24 h, they are demolded, then stored in a water bath at a constant temperature of 20 °C. The samples will be crushed after 7, 15, 28, 60, and 90 days to evaluate their compressive strength (CS).

Two different types of specimens were manufactured:

- Figure 2a: mortar prisms (4 × 4 × 16) containing BA to study their pozzolanic activity (BA is used as a substitute for normalized sand);
- Figure 2b: compacted test tubes of the BA specimens for the other characterization tests.

### 2.4. Treatment of Bottom Ash with Lime and Sand 

#### 2.4.1. Overview of the Experimental Approach Adopted for the Treatment of BA

In order to improve the properties of the BA and with the intention of using them as foundation materials, they were subjected to a lime and sand treatment. The experimental approach adopted to establish mixing dosages and also to model the results is the design of experiments method [6]. The experimental design consists of selecting and ordering tests to identify the effects of parameters (factor *X_i_*) on the desired properties (response *Y_i_*) of a material. These are statistical methods using simple mathematical concepts. The implementation of this method involves the following main steps:

1. Identification of the properties to be studied (responses);
2. Identification of the parameters (factors) that influence these responses;
3. Definition of the ranges of variation of each factor, with two levels of extreme variation (±1);
4. Carrying out the experiments planned by the model;
5. Analysis of the results (answers) and mathematical modeling.

#### 2.4.2. Experimental Plan Adopted for the Treatment of BA

The final material studied is a mixture of BA treated with lime and sand. The factors considered to influence the properties of this mixture are:

- The percentage of BA;
- The water content noted as *W*;
- The percentage of lime noted as *L*;
- The percentage of calcarenite sand noted as *S*.

Other factors can be mentioned, such as the nature of the lime and sand, the compaction energy, the temperature during implementation, the mixing, and the storage conditions of the specimens. In this study, all of these were kept constant. In addition, the total solid mass of the mixture satisfies the following condition, given by Equation (1):

(1) ∑(% BA+% S+% L)=100%

The responses studied to evaluate the possibility of using the mixture as a foundation layer are:

- Compressive strength at 60 days noted as *CS_60_*;
- Dry density noted as *γ_d_*;
- Swelling noted as *G*;
- Dry CBR index noted as *CBR_d_*;
- The CBR index after immersion noted as *CBR_w_*.

There are several types of experimental design [18]. In this study, we opted for a centered composite factorial design composed of the following elements:

- A two-level factorial plan (−1, +1);
- An experimental point located in the center of the field of study;
- Experimental points located on the axes of each of the 3 factors. These points belong to the interval (−α, +α).

The experimental design adopted will make it possible to evaluate the effects of the three factors at five different levels: −α, −1, 0, 1, and +α. The value of α is calculated as follows: α = NP^1/4^, with NP the number of points of the factorial plane. In our study this leads to the following value, Equation (2):

(2) $$\alpha =8\times \frac{1}{4}=\left(23\right)\times \frac{1}{4}=1.683$$

The three main factors Lime (*L*), Water (*W*), and Sand (*S*) are associated with the reduced centered variables: *A, B* and *C*. The levels +α and −α are assigned to the extreme values of *A, B* and *C*. The levels −1, 0, and +1 are obtained by linear interpolation (see Table 1). The relationships between the reduced centered variables and the real variables are given by Equation (3)–(5):

(3) $$A=\frac{L-2,5}{1.485}$$

(4) $$B=\frac{W-22}{0.367}$$

(5) $$C=\frac{S-12,5}{7.427}$$

To obtain statistically representative results, each test is performed with three replicates. The number of specimens manufactured is distributed as follows:

- 24 = 2^3^ × 3 tests in the factorial design, in which the factors were adjusted to the −1 and +1 levels.
- 18 = (2 × 3) × 3 axial tests in which the factors were adjusted to the ±α levels, to estimate the quadratic effect of the different parameters on the responses.
- 6 = 2 × 3 center tests for model verification and determination of the experimental error.

In order to obtain results for *CS_60_, γ_d_*, *G, CBR_d_* and *CBR_w_* this study required (24 + 18 + 6) × 4 = 192 cylindrical specimens, see Figure 2b. All the tests are represented by the matrix included in Table 2.

## 3. Results

### 3.1. Chemical and Mineralogical Characteristics of Bottom Ash

The elemental composition of the studied BA and those found in the literature are given in Table 3. The results show that the BA mainly consists of silicon dioxide (SiO_2_), aluminum oxide Al_2_O_3_ and ferric oxide (Fe_2_O_3_) with small amounts of calcium oxide, magnesium oxide, and potassium hydroxide. The sum of the percentages of the elements SiO_2_, Fe_2_O_3_, and Al_2_O_3_ exceeds 80% of the total mass, while the percentage of CaO remains low. Therefore, BA can be classified as a silico-aluminous material (class F) according to the ASTM 225 standard [25]. This table also shows that the BA used in this study has a chemical composition that is very close to the BA found in literature [2,28,29]. In Table 1 of Reference [2] an overview is included showing the chemical analyses from different studies.

**Figure 3:**
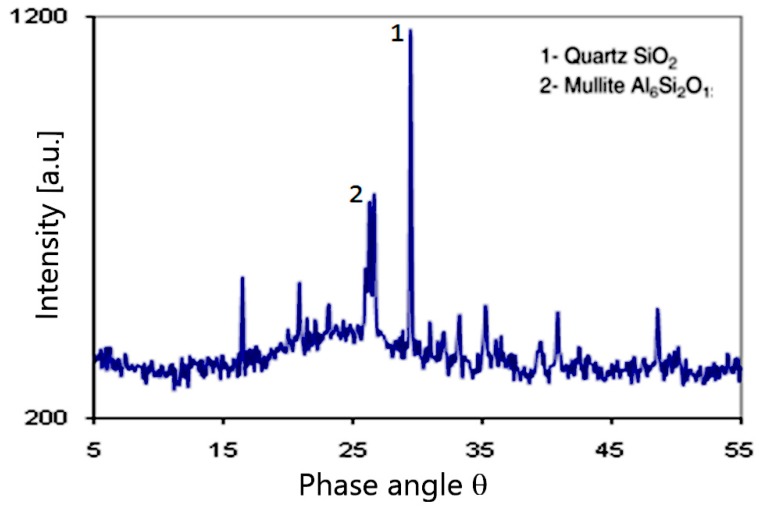
Mineralogical composition of bottom ash (BA) using X-ray diffraction.

The mineralogical composition of the BA, determined with X-Ray diffraction, reveals the existence of two peaks, see Figure 3. The first is quartz and the second is mullite. These two mineralogical phases are the same as those found for some BA studied in literature [28,29]. 

### 3.2. Physical Characteristics of Bottom Ash

The visual observation of the BA shows that it has a gray color and a porous texture. Its absolute density is 1760 kg/m^3^. This value is low compared to natural aggregates such as silica sand which has an absolute density of 2600 kg/m^3^ [28], and BA tested in [9] with an absolute density of 2560 kg/m^3^, but very close to the value for BA of 1880 kg/m^3^ reported in [30]. In Table 2 from Reference [2] values ranging between 1390 and 2470 kg/m^3^ are shown. The porous texture is the same as that observed for BA produced in other countries [2,28]. As discussed in Section 2.3, the cleanliness of the BA is verified by two tests: the sand equivalent (SE) and the methylene blue. The result of the first test is SE = 78%, which classifies the BA as clean sand with a low percentage of fine clay, which is ideal for construction work [19]. The second test yielded a methylene blue value of 0.5. This value is less than 1.5, which represents the threshold for silty sandy soils in sandy loam soils. It can be deduced from these two tests that the BA is clean and insensitive to water which encourages their use in the road domain. 

**Figure 4:**
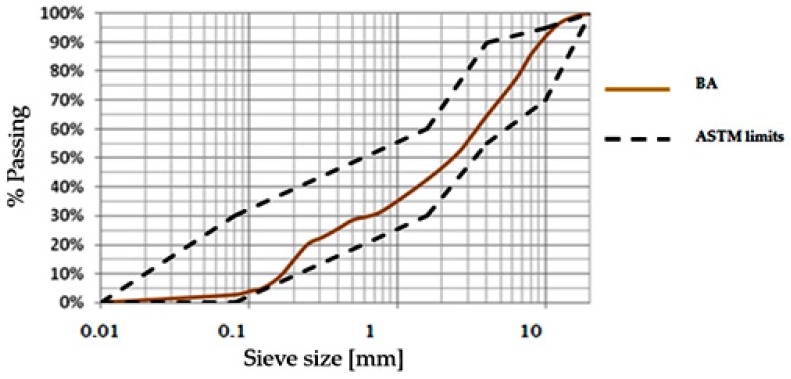
Granulometric curve of JLEC BA.

The granulometric study of the BA (see Figure 4) shows that their particle size distribution (PSD) is very close to the sand. It also shows that their granulometric curve is located within the ASTM standard granular limits for BA used as foundation layer [31].

### 3.3. Compaction of Raw Bottom Ash 

The Proctor test shows that the maximum density of the BA is 1.26 kN/m^3^, and is obtained at a water content of 21.6%. This high value of the Proctor moisture content indicates a high porosity of the material. Moreover, this value of the optimum Proctor of the BA is very low compared to conventional materials such as clay and sand whose dry density is respectively between 16 and 21 kN/m^3^. Hence the need to treat the BA in order to improve this characteristic.

The *CBR_d_* value obtained is 35%, exceeding the min. standard value of 20%. The BA is then classified in the lift class AR3 [26]. On the other hand, the punching test performed on a BA sample immersed for four days gives a *CBR_w_* value of 58%, which exceeds the *CBR_d_* value of 35%. This increase in lift is significant and should be exploited in the road sector.

Finally, the recorded value of the swelling (*G*) was zero. This result shows that the BA does not represent any risk of swelling and promotes its use in the road construction.

### 3.4. Pozzolanic Activity of BA

**Figure 5:**
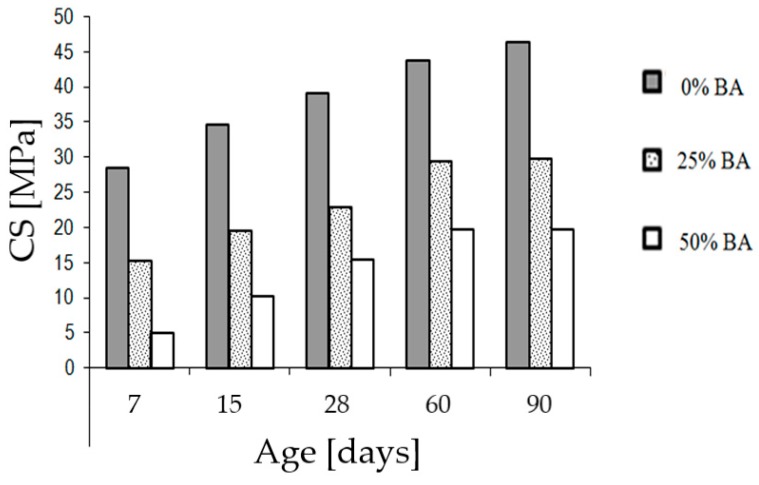
Compressive strength of mortar containing bottom ash.

The evaluation of the mechanical behavior is performed by determining the compressive strength of mortar prisms, see Figure 2a, prepared from a mixture of CPJ 45 cement, water and BA as a partial substitute for the standardized sand. The results show that the compressive strength of the prisms containing BA is, at any age, adversely influenced by the use of BA, see Figure 5. The decrease in resistance becomes very important for 50% of BA and especially at a young age. These results are in the same order of magnitude as those found by other researchers [8,29]. They also found that the decrease of the compressive strength of mortar prisms containing BA can be explained by the dominant role of porosity, which is inversely proportional to compressive strength. In fact, the substitution of the most resistant material (standardized sand) by the weaker and more porous material (BA) increases the fraction of the pores of the mortar which decreases the compressive strength.

As shown in Figure 5, an increase of the pozzolanic potential of the BA, especially at 60 and 90 days, becomes apparent. Therefore, during the treatment of the BA, the evolution of the compressive strength will be studied at 60 days instead of 28 days, because at this age the pozzolanic power of the BA is important and the compressive strength is significant. The composition of each mortar mixture and the values of their compressive strength at different ages (one sample per test) are given in Table 4.

### 3.5. Property of the BA-Lime-Sand Mixture

#### 3.5.1. Presentation of Experimental Results on Treated BA

The average results and standard deviation of all tests performed on lime and sand treated BA are given in Table 5. Each value represents the average of three tests. These results show that in addition to the specific weight of the BA, mechanical properties such as compressive strength, and the dry and wet CBR indices are positively influenced by the addition of lime and sand. This result may be due to the increased compactness thanks to the addition of the sand or the activation of a pozzolanic reaction after the addition of lime. We also note that, for each treated BA mixture (TBA), CBR values after submersion (*CBR_w_*) are higher than those of dry CBR (*CBR_d_*) which represents a lift gain that cannot be found for conventional materials. This result is very interesting in terms of lift gain and reveals the importance of the use of BA in the road domain. Moreover, the swelling remains very low, as we wish, which encourages us to use the treated BA without risk of volume instability. The dry density was determined from its wet density using Equation (6):

(6) $$\gamma_{d}=\frac{\gamma_{w}}{1+W}$$

#### 3.5.2. Modeling Responses

For each response studied, we proceed to the modeling of the results using the Design-Expert software. This starts with the analysis of the variance, which allows the determination of the influencing factors and the elimination of the insignificant factors. Then a model combining each response to influential factors is developed. From this fact, a mathematical function connecting the response to the factors is proposed by the model. The modeling equations for the responses (*CBR_d_, CBR_w_, γ_d_, G* and *CS_60_*) are expressed in terms of the percentages of lime (*L*), water (*W*) and sand (*S*). We thus obtain the following Equation (7)–(11):

(7) $$CS_{60}{}^{2}=1395.96-21.41\times L-126.8\times W+0.14\times S+1.08\times L\times W-0.06\times L\;\times S-0.08\times L^{2}+2.88\times W^{2}$$

(8) $$\gamma_{d}{}^{3}=12525.76-2892.09\times L-513.29\times W-442.31\times S+133.3\times L\times W\;+20.66\times W\times S$$

(9) $$CBR_{d}{}^{-0.85}=-0.04+\left(13.42\times L+27.28\times W+2.78\times S+0.69\times L\times S-0.52\;\times L2-0.19\times S2\right)\times 10^{-4}$$

(10) $$CBR_{w}{}^{2}=5397.02+588.98\times L-2246.85\times W$$

(11) $$G^{0.65}=2.19+0.04\times L-0.1\times W+0.02\times S-6.68\times 10^{-4}\times S^{2}$$

#### 3.5.3. Optimal Predicted Formulations

Taking into account the prerequisites of using BA as a foundation material, the models obtained make it possible for us to propose two optimal formulations, predicted from the actual test results. 

The first optimal formulation is technical and takes into consideration the following requirements:

1. Maximize *CS_60_*, *CBR_w_*, and *CBR_d_*.
2. Minimize swelling (*G*) and percentages of lime and added sand.

The first optimal formulation is obtained with 2% of lime, 22.4% of water and 5% of sand. The predicted results for this first formulation are:

- *CBR_d_* = 62%
- *CBR_w_* = 68%
- Swelling (*G*) = 0.05%
- *CS_60_* = 2.05 MPa

The second variant is economical. It aims to reduce the cost by minimizing the processing materials while keeping acceptable mechanical properties and it takes into consideration the following requirements:

1. Minimize swelling (*G*)
2. Minimize the cost
3. CS60 > 1.5 MPa

The second optimal formulation is obtained with 0.3% of lime, 22.4% of water and 20% of sand. The predicted results for the second formulation are:

- CBR_d_ = 74%
- CBR_w_ = 61%
- Swelling (*G*) = 0.01%
- *CS_60_* = 1.5 MPa

Both optimal formulations are acceptable although the compressive strength is less than 3 MPa. The CBR-values of both variants are sufficient for the proposed application and it is the major parameter taken into consideration for the dimensioning of this kind of structure. Moreover, the compressive strength parameter is studied in our case just to ensure the efficiency of the treatment. Higher compressive strength values are required for rigid structures (with concrete) which is not the case for our proposed pavement structure.

### 3.6. Use of Treated BA in Road Pavement

**Figure 6:**
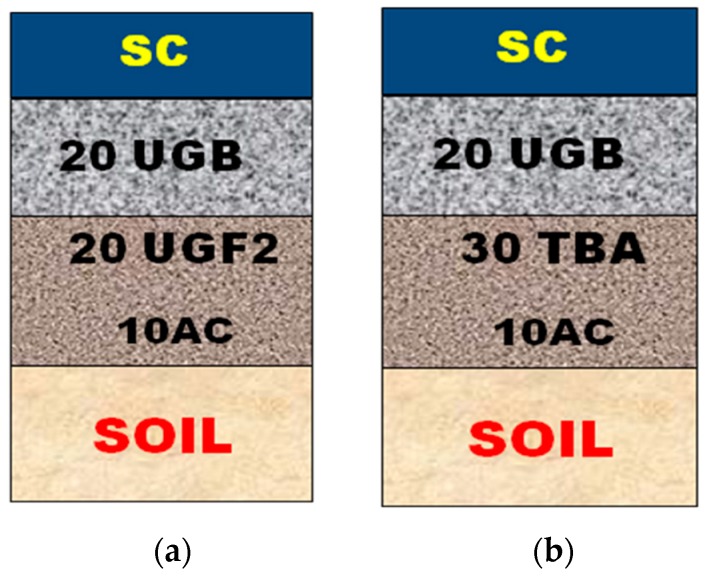
(**a**) Conventional pavement structure, (**b**) Pavement structure based on TBA.

Characterization of the BA shows that they have very satisfactory characteristics: their granulometric curve is located inside the granular limits of BA valued in the roads. They are also clean and are insensitive to water. Moreover, their treatment with lime and sand has clearly improved their mechanical properties: compressive strength (*CS_60_*), dry and wet CBR indices (*CBR_d_*, *CBR_w_*) and dry density *γ_d_* all reached satisfactory levels after this treatment, while the swelling *G* remains very low. These results encourage us to use the BA in the foundation layer of a rural road, e.g. near the city of El Jadida in Morocco, thus minimizing transportation costs. The design method adopted is the classic method of the New Pavement Structure Catalog [19]. The parameters taken into consideration in the design of this route are the traffic which is class TPL3, the climate which is considered wet for the rural area of El Jadida, and the supporting ground which has a bearing capacity class of ST1. The conventional pavement structure proposed by the catalog, presented in Figure 6a, consists of 10 cm AC + 20 cm UGF2 + 20 cm UGB + SC, with:

- AC: Anti-Contamination layer;
- UGF2: Untreated Gravel for Foundation layer type 2;
- UGB: Untreated Gravel for the base layer;
- SC: Superficial Coating.

In the proposed pavement structure, we used the TBA (BA treated with lime and sand) as a foundation layer material instead of the UGF2. However, taking into account the modest CS of the TBA, we propose an increase in the thickness of the foundation layer, 30 cm of TBA instead of 20 cm of UGF2. The new proposed variant, see Figure 6b, consists of 10 cm AC + 30 cm TBA + 20 cm UGB + SC.

## 4. Conclusions

The results obtained in this study show that, despite their low density, the BA studied has interesting properties that can be used in order to promote their value in road engineering, particularly in the foundation layer. The results of this study are summarized as follows:

1. The Jorf Lasfar bottom ash BA is class F. It has a significant pozzolanic power, which favors their treatment with a hydraulic binder.
2. The lime treatment of the BA significantly improves their properties, which are: the compressive strength (*CS_60_*), the dry density, and the CBR indices. The best mechanical performances are obtained for a mixture with 4% of lime.
3. The use of calcarenite sand increases the dry density of the treated BA mixture (TBA) compared to that of the original BA. It is deduced that sand corrects the porous texture of BA by increasing their compactness. The maximum density is registered for 25% of sand.
4. According to the performance obtained after the treatment of BA with lime and sand, we propose their use as a road material in the rural roads of the city of El Jadida in Morocco. In the proposed pavement structure, we used the TBA as a foundation layer instead of the UGF2 (untreated granulate for foundation layer type 2). This valorization makes it possible, on the one hand, to provide a cheaper ecological rural road network and, on the other hand, to find a sustainable solution for landfilling the BA.

The results of this research constitute a first step forward in the field of valorization of BA in Moroccan pavements. However, the stakes remain very important if we take into account the millions of tons of this waste produced worldwide each year. Further research should include an analysis of leaching from the treated BA mixture and preferably a complete Life Cycle Assessment. If these studies are positive, the proposed pavement structure, containing BA, should be tested further, under real traffic conditions, in order to monitor its behavior and ensure its durability.

## Figures and Tables

**Table 1:** Factor Levels of the Centered Composite Plane.

Factor Levels	−α = −1.683	−1	0	+1	+α = +1.683
Lime dosage (%)	0	1.015	2.5	3.985	5
Water dosage (%)	21.4	21.762	22	22.238	22.6
Sand dosage (%)	0	5.073	12.5	19.927	25

**Table 2:** Matrix of the tests of the composite centered factorial plan adopted for the treatment of bottom ash (BA).

Tests	Lime Dosage	Water Dosage	Sand Dosage
	A	B	C
1	−1	−1	−1
2	1	−1	−1
3	−1	1	−1
4	1	1	−1
5	−1	−1	1
6	1	−1	1
7	−1	1	1
8	1	1	1
9	−α	0	0
10	+α	0	0
11	0	−α	0
12	0	+α	0
13	0	0	−α
14	0	0	+α
15	0	0	0
16	0	0	0

**Table 3:** Elemental composition (%) of bottom ash and used cement.

Chemical Element	CaO	SiO_2_	Fe_2_O_3_	Al_2_O_3_	K_2_O	Na_2_O	P_2_O_5_	SO_3_	MgO	Free CaO
(JLEC) BA	1.9	52.1	8.9	23.3	1.9	0.4	0.1	<1	0.9	0.3
BA [28]	4.2	50.5	10.9	27.6	0.8	0.6	0.2	0.1	1.2	-
BA [29]	7.0	46.1	5.8	23.7	1.2	0.7	-	-	1.2	-
Cement CPJ45	63.0	17.0	3.0	5.0	1.2	-	-	3.3	2.3	-

**Table 4:** Composition of the mortar mixture and compressive strength at different ages.

Type	Composition (g)				CS (MPa) at Different Ages (days)				
Mortar	Cement	Water	Sand	BA	7	15	28	60	90
Reference	450	225	1350	0	28.5	34.7	39	43.8	46.4
25% BA	450	225	1012.9	337.5	15.1	19.4	22.8	29.3	29.7
50% BA	450	225	675	675	5	10.3	15.4	19.7	19.8

**Table 5:** Average results of tests carried out on treated BA.

Test	Levels of Factors			Average Responses and Standard Deviation				
Number	Lime (%)	Water (%)	Sand (%)	*CBR_d_* (%)	*CBR_w_* (%)	*γ_d_* (kN/m^3^)	*G* (%)	*CS_60_* (MPa)
1	1	21.6	5	79.0 (6.5)	98.3 (9.0)	11.7 (0.20)	0.06 (0.03)	1.10 (0.09)
2	4	21.6	5	82.9 (9.8)	107.0 (15.8)	11.5 (0.03)	0.15 (0.02)	2.36 (0.08)
3	1	22.4	5	58.6 (1.5)	73.0 (9.0)	11.0 (0.11)	0.03 (0.00)	1.63 (0.03)
4	4	22.4	5	75.2 (6.9)	86.0 (14.3)	11.5 (0.20)	0.13 (0.02)	2.84 (0.02)
5	1	21.6	20	74.0 (3.7)	90.7 (6.6)	11.6 (0.10)	0.12 (0.01)	1.65 (0.07)
6	4	21.6	20	80.4 (5.1)	72.7 (3.1)	11.3 (0.03)	0.14 (0.01)	1.74 (0.00)
7	1	22.4	20	74.5 (5.3)	78.0 (1.6)	11.5 (0.05)	0.04 (0.00)	1.87 (0.05)
8	4	22.4	20	66.3 (8.1)	85.7 (6.6)	11.9 (0.05)	0.11 (0.02)	2.75 (0.08)
9	0	22.0	12.5	68.7 (5.2)	36.3 (5.7)	10.7 (0.12)	0.02 (0.00)	0.43 (0.04)
10	5	22.0	12.5	79.2 (0.5)	83.3 (6.9)	11.8 (0.15)	0.12 (0.02)	2.61 (0.13)
11	2.5	21.4	12.5	66.3 (3.9)	77.3 (13.7)	11.0 (0.13)	0.11 (0.02)	2.02 (0.06)
12	2.5	22.6	12.5	59.4 (4.5)	65.3 (10.3)	11.7 (0.08)	0.02 (0.00)	2.48 (0.14)
13	2.5	22.0	0	70.0 (4.9)	58.3 (6.2)	10.6 (0.09)	0.02 (0.00)	1.97 (0.02)
14	2.5	22.0	25	75.0 (6.1)	59.0 (2.9)	12.1 (0.15)	0.01 (0.00)	1.87 (0.03)
15	2.5	22.0	12.5	62.8 (1.4)	69.0 (11.0)	11.4 (0.02)	0.12 (0.02)	2.08 (0.08)
16	2.5	22.0	12.5	62.5 (0.4)	64.3 (2.5)	11.3 (0.06)	0.12 (0.02)	2.06 (0.04)
